# Pneumonia diagnosis performance in the emergency department: a mixed-methods study about clinicians’ experiences and exploration of individual differences and response to diagnostic performance feedback

**DOI:** 10.1093/jamia/ocae112

**Published:** 2024-05-25

**Authors:** Jorie M Butler, Teresa Taft, Peter Taber, Elizabeth Rutter, Megan Fix, Alden Baker, Charlene Weir, McKenna Nevers, David Classen, Karen Cosby, Makoto Jones, Alec Chapman, Barbara E Jones

**Affiliations:** Department of Biomedical Informatics, University of Utah Spencer Fox Eccles School of Medicine, Salt Lake City, UT 84108, United States; Department of Internal Medicine, Division of Geriatrics, University of Utah Spencer Fox Eccles School of Medicine, Salt Lake City, UT 84132, United States; Salt Lake City VA Informatics Decision-Enhancement and Analytic Sciences (IDEAS) Center for Innovation, Salt Lake City, UT 84148, United States; Geriatrics Research, Education, and Clinical Center (GRECC), VA Salt Lake City Health Care System, Salt Lake City, UT 84148, United States; Department of Biomedical Informatics, University of Utah Spencer Fox Eccles School of Medicine, Salt Lake City, UT 84108, United States; Department of Biomedical Informatics, University of Utah Spencer Fox Eccles School of Medicine, Salt Lake City, UT 84108, United States; Salt Lake City VA Informatics Decision-Enhancement and Analytic Sciences (IDEAS) Center for Innovation, Salt Lake City, UT 84148, United States; Department of Emergency Medicine, University of Utah Spencer Fox Eccles School of Medicine, Salt Lake City, UT 84108, United States; Department of Emergency Medicine, University of Utah Spencer Fox Eccles School of Medicine, Salt Lake City, UT 84108, United States; Department of Family and Preventive Medicine, Division of Physician Assistant Studies, University of Utah Spencer Fox Eccles School of Medicine, Salt Lake City, UT 84108, United States; Department of Biomedical Informatics, University of Utah Spencer Fox Eccles School of Medicine, Salt Lake City, UT 84108, United States; Department of Internal Medicine, Division of Epidemiology, University of Utah Spencer Fox Eccles School of Medicine, Salt Lake City, UT 84108, United States; Department of Internal Medicine, Division of Epidemiology, University of Utah Spencer Fox Eccles School of Medicine, Salt Lake City, UT 84108, United States; Department of Emergency Medicine, Cook County Hospital, Rush Medical College, Chicago, IL 60612, United States; Salt Lake City VA Informatics Decision-Enhancement and Analytic Sciences (IDEAS) Center for Innovation, Salt Lake City, UT 84148, United States; Department of Internal Medicine, Division of Epidemiology, University of Utah Spencer Fox Eccles School of Medicine, Salt Lake City, UT 84108, United States; Department of Population Health Sciences, University of Utah Spencer Fox Eccles School of Medicine, Salt Lake City, UT 84108, United States; Salt Lake City VA Informatics Decision-Enhancement and Analytic Sciences (IDEAS) Center for Innovation, Salt Lake City, UT 84148, United States; Department of Internal Medicine, Division of Pulmonology, University of Utah Spencer Fox Eccles School of Medicine, Salt Lake City, UT 84108, United States

**Keywords:** diagnosis, individual differences, sociotechnical, usability, feedback

## Abstract

**Objectives:**

We sought to (1) characterize the process of diagnosing pneumonia in an emergency department (ED) and (2) examine clinician reactions to a clinician-facing diagnostic discordance feedback tool.

**Materials and Methods:**

We designed a diagnostic feedback tool, using electronic health record data from ED clinicians’ patients to establish concordance or discordance between ED diagnosis, radiology reports, and hospital discharge diagnosis for pneumonia. We conducted semistructured interviews with 11 ED clinicians about pneumonia diagnosis and reactions to the feedback tool. We administered surveys measuring individual differences in mindset beliefs, comfort with feedback, and feedback tool usability. We qualitatively analyzed interview transcripts and descriptively analyzed survey data.

**Results:**

Thematic results revealed: (1) the diagnostic process for pneumonia in the ED is characterized by diagnostic uncertainty and may be secondary to goals to treat and dispose the patient; (2) clinician diagnostic self-evaluation is a fragmented, inconsistent process of case review and follow-up that a feedback tool could fill; (3) the feedback tool was described favorably, with task and normative feedback harnessing clinician values of high-quality patient care and personal excellence; and (4) strong reactions to diagnostic feedback varied from implicit trust to profound skepticism about the validity of the concordance metric. Survey results suggested a relationship between clinicians’ individual differences in learning and failure beliefs, feedback experience, and usability ratings.

**Discussion and Conclusion:**

Clinicians value feedback on pneumonia diagnoses. Our results highlight the importance of feedback about diagnostic performance and suggest directions for considering individual differences in feedback tool design and implementation.

## Background and significance

Diagnosis is one of the major tasks in clinical practice, but misdiagnosis is a major source of medical error in emergency department (ED) and hospital settings.^1^^,^^2^ Improving diagnostic accuracy through measurement and feedback is increasingly recognized and called for by key quality organizations including the National Academy of Medicine, Joint Commission, and National Quality Forum.^3–7^ Electronic health record (EHR)-derived measurements and computerized feedback tools have been shown to improve diagnostic accuracy^8^^,^^9^ and offer low burden solutions to consistently identify learning opportunities and promote an open culture of safety in High Reliability organizations.^10–12^ However, such tools may present new challenges that are poorly understood.

Diagnosis is a complex process of information seeking, clinical reasoning, and decision-making^13^ and can be uncertain, subjective, and dynamic, raising new issues with measurement.^14–16^ On one hand, measures that are properly connected to clinicians’ goals to provide high-quality patient care can engage and motivate clinicians.^17^ However, measures of performance can also thwart an open culture of safety if misused or not supported by practice environments that support acknowledging and learning from mistakes.^18^^,^^19^

Interpreting feedback is a complex process influenced by individual characteristics, context, and experience.^20^^,^^21^ Exploring individual differences in beliefs including motivational theories, such as mindset^22^ and failure beliefs are crucial to better understand clinicians’ responses to feedback.^23^^,^^24^ Believing intelligence is stable (“fixed mindset”) compared to the belief that intelligence to mutable and relating to experience (“growth mindset”) may influence the experience of feedback as useful, uncomfortable, or even threatening.^22^^,^^23^^,^^25–27^ This relates to the individuals’ underlying mental model of the connection between intelligence and performance such that poorer performance may point to being a less intelligent person and thus potentially more distressing for those with fixed mindsets. Failure attributions relate to an individuals’ concern that failure will upset important others, lead to personal devaluing of ones’ own capabilities, be shameful or embarrassing, or produce other negative consequences.^28–30^ Understanding how beliefs such as these contribute to learning and improvement is critical if we are to create usable, meaningful feedback tools and performance measures that support a continuous learning healthcare system culture.^24^^,^^25^ Understanding beliefs is consistent with the adult learning theory-based principles of problem-based learning and self-directed learning, but the mindset and failure attributions are tailored specifically to potential challenges in design of feedback tools.^31^

Pneumonia is an ideal clinical diagnosis for disentangling complex factors in diagnostic accuracy. A common syndrome with high consequences,^32^^,^^33^ pneumonia is a frequently missed diagnosis and a common target of malpractice claims.^34–36^ Up to 30% of hospitalized patients who receive a clinical diagnosis of pneumonia lack confirmative positive chest imaging.^36–42^ The majority of pneumonia cases are diagnosed in the ED, where clinicians encounter exceptionally complex patients to evaluate, frequent interruptions, time pressure, and substantial cognitive load, which can all contribute to inaccurate initial diagnoses.^43^^,^^44^ Currently, clinicians rarely receive feedback about diagnosis, constituting missed opportunities for self-assessment and diagnostic skills improvement.^6^^,^^45^ Prior work by our team showed over half of all cases of pneumonia demonstrated discordances between the ED diagnosis and discharge diagnosis.^46^ Diagnostic discordances may not always represent individual clinician error, but their measurement is an efficient way to identify cases for review to improve clinician performance.

The purpose of this mixed-methods study was to characterize the process of pneumonia diagnosis in the ED and the clinicians’ experience of receiving feedback about their performance using a tool we developed and designed for ED clinicians, “Dx-Connect.” Dx-Connect displays patient data from the EHR and measures of diagnostic performance at the clinician level, measured by concordance/discordance between the initial diagnosis, radiology report, and discharge documentation, thus connecting the provider to ultimate diagnostic and clinical outcomes of their patients. We examined the providers’ responses to this feedback with semistructured interviews and surveys.

## Methods

### Recruitment and ethics

We identified a cohort of patient hospitalizations from the ED at an academic medical center within 12 months preceding the study (October 1, 2020, to September 30, 2021) with an initial or discharge diagnosis of pneumonia identified by a previously validated approach that combines International Classification of Disease (ICD) 10 diagnosis codes and natural language processing (NLP).^46^ ED clinicians with at least 10 pneumonia cases (n = 123) were eligible for participation and received a department-wide email with general information about the study and invitation to participate. We accepted 1 volunteer participant who responded to the general email and purposively sampled an additional 15 participants for email invitation into the interview and survey portion of the study. Purposive sampling was based on age, gender, and type (physician vs advanced practicing clinician) to increase representativeness of the sample of interviewees. A consent cover letter was presented at the outset of the interview. All study procedures were reviewed and approved by the University of Utah Institutional Review Board (IRB # 00136521).

## Procedures

Using a sequential mixed-methods design we (1) analyzed diagnostic discordance data for ED clinicians and prepared a visualization of these data for display in prototype Dx-Connect, (2) interviewed ED clinicians about diagnosing pneumonia in the ED context and their reactions when exploring the prototype feedback tool, and (3) surveyed clinicians to assess comfort with their personal performance feedback, mindset about intelligence, comfort with failure, and feedback tool usability.

### Quantitative analysis of diagnostic performance

Using the EHR from the University of Utah (UU), we developed, tested, and implemented measures of diagnostic accuracy for ED providers caring for patients admitted to the hospital with pneumonia between January 1, 2015, and March 31, 2022. We identified discordances between the initial/ED diagnosis, chest imaging diagnosis, and the final/discharge diagnosis of pneumonia by combining diagnosis codes with natural language (NLP) of clinical text from ED documents, chest imaging reports, and discharge summaries.^47^ Treating the initial/ED diagnosis as a “test” diagnosis and the discharge diagnosis as an imperfect reference standard and “true,” we classified each case evaluated by the clinician as either “true positive” (concordance between ED diagnoses and discharge diagnoses [+/+], “false positive” [+/−], “false negative” [−/+], or “true negative” [−/−]). We also classified discordance or concordance between initial positive cases with the diagnosis of pneumonia within the chest imaging report. (“false positive” [+/−] or “true positive” [+/+]). For each participating provider and the entire department, we summarized annual and quarterly: (1) positive predictive value (PPV) against chest image as the percent of ED diagnoses for pneumonia that also had a positive chest image; (2) PPV against discharge as the percent positive ED diagnoses with a discharge diagnosis for pneumonia, and (3) the sensitivity against discharge diagnosis as the percent of positive discharge diagnoses of pneumonia that also had an initial positive ED diagnosis.

### Prototype feedback tool

**Figure 1.**
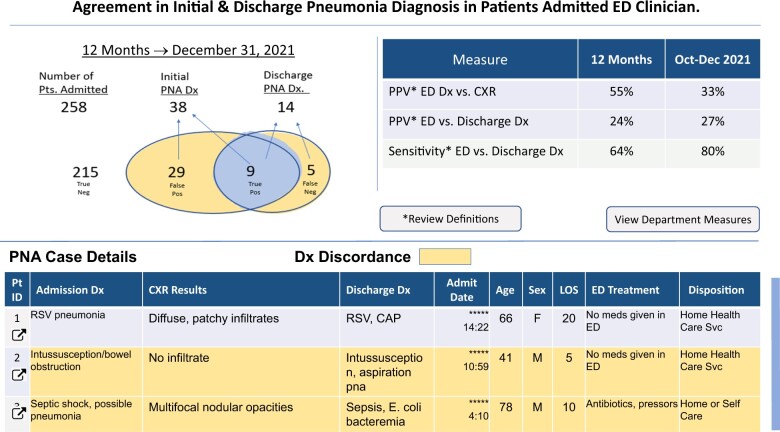
Agreement in initial and discharge pneumonia diagnosis in patients admitted ED clinician.

Dx-Connect incorporates EHR data and displays clinician-level diagnosis data for pneumonia cases that were hospitalized from the ED by the participating clinician (Figure 1).

**Figure 2.**
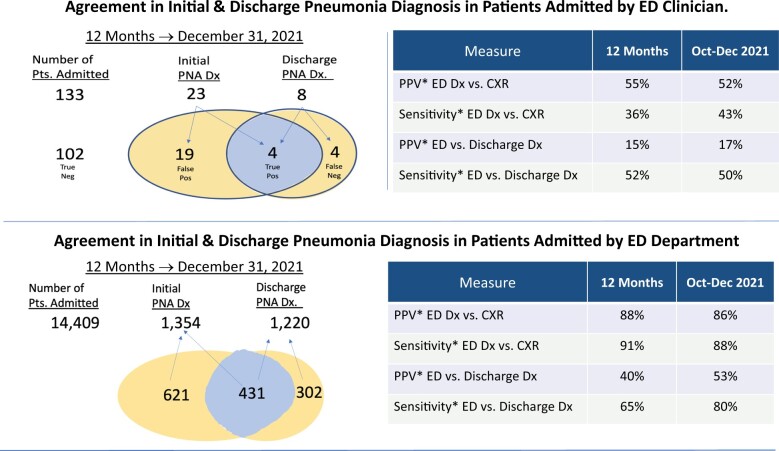
Agreement in initial and discharge pneumonia diagnosis in patients admitted by ED clinician.

**Figure 3.**
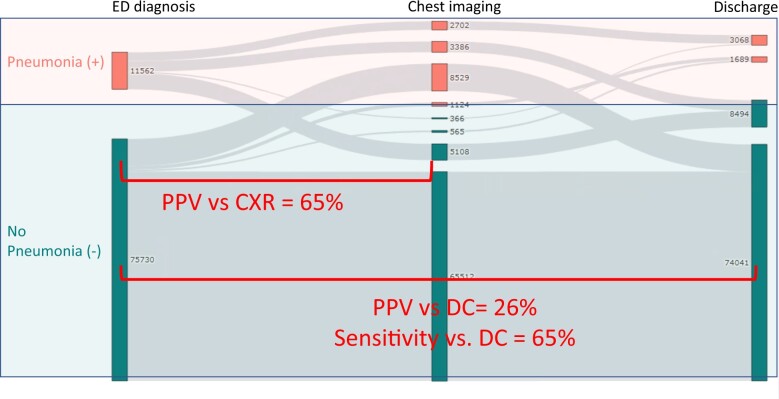
Diagnostic Accuracy Assessment for Emergency Department. ED diagnoses of Pneumonia are shown in upper left, and discharge diagnoses in upper right. The Chest imaging in center displays the number of diagnoses confirmed/disconfirmed with chest imaging.

Additional features included (1) a Venn Diagram displaying the total number of pneumonia encounters with an initial diagnosis by that clinician and the number of concordant and discordant cases, (2) quarterly and annual positive predictive value and sensitivity, and (3) a table with select patient case details. Case clinical notes, discharge summaries, and chest X-ray reports (with diagnosis terms highlighted by NLP) were accessible through links embedded in the table. We also created a method for clinicians to compare themselves to their peers as seen in the “View Department Measures Button” (see Figure 2). Clicking this button generated a view of relative performance compared to other clinicians in the ED in which they worked for concordant diagnoses (see Figure 3).

### Interview

We designed a semistructured interview, with input from an ED physician, pulmonary critical care physician, behavioral scientist, and an informaticist, that focused on pneumonia diagnostic experiences, presentation of individual diagnostic performance feedback, and exploration of how the feedback would be used (see Supplementary material). The interview followed a cognitive task analysis (CTA) approach prompting clinicians to describe a specific case of pneumonia diagnosis in the ED to characterize the cognitive process of pneumonia diagnosis in the ED.^48^^,^^49^ Interviewers described themselves as researchers engaged in development and testing of the feedback tool. We then presented the feedback tool, described above, to the provider and asked questions surrounding their understanding of the measure and their reactions to their feedback.

### Survey measures

Following the interview, participants were provided with a link to complete a survey online via Qualtrics (Qualtrics LLC, United States). The survey included a 3-item *Mindset* questionnaire assessing beliefs related to a growth mindset and implicit theories of intelligence.^50^^,^^51^ The *Performance Failure Attribution Inventory* was used to assess beliefs about failure.^52^ A single item assessed *Comfort with Feedback*. Participants also completed the *System Usability Scale* (SUS)^53^ a usability assessment applied to thousands of informatics tools with a range of 0-100 with higher scores indicating increased usability. The mean score across tools is 68.^54^

## Analysis

### Quantitative analysis of EHR data

Department-level PPV against chest image, PPV against discharge, and sensitivity against discharge diagnosis were summarized.

### Qualitative thematic analysis

We used a thematic analysis approach to code the transcripts of the semistructured interviews with the study team experts who developed the interview using the qualitative analysis software program NVIVO (QSR International, Australia).^55–57^ We reviewed 5 transcripts together and assigned precodes—categorizations based directly on participants’ statements. We then developed a code book with code names, definitions, and sample quotes. The remaining 6 transcripts were coded independently by 2 coders per transcript with discrepancies resolved by full coding team consensus. Specific codes were grouped into themes through iterative discussion and then reviewed by two additional ED physicians (E.R. and M.F.). Interview participants were invited to review and comment on the thematic results as a member checking procedure for validation.^58^ All interviewees received an email with a summary of findings. We received no comments back from participants about the findings.

### Descriptive survey analysis

We conducted exploratory, descriptive analysis of survey data using SPSS (SPSS Inc., United States).^59^ Median values are more interpretable than means in small samples; thus, we split the Mindset Scale and the Failure Attribution Scale at the sample median, to explore the relationship with comfort with receiving feedback and usability ratings. Then, we conducted cross tabs exploration and graphically displayed how participants at above and below sample median level of fixed vs growth intelligence levels and failure attribution levels were distributed across feedback comfort and the System Usability Scale (SUS).

## Results

### Quantitative analysis

Diagnostic discordance in pneumonia was high occurring in over half of all pneumonia hospitalizations (Figure 2). Among patients with an initial ED diagnosis of pneumonia, the PPV against chest image (percent with a positive chest image) was 65%, and PPV against discharge (percent with positive discharge diagnosis for pneumonia) was 26%. Among those patients with a discharge diagnosis of pneumonia, 65% had an initial ED diagnosis of pneumonia.

A total of 11 interviews and 9 surveys were completed.^60^ Clinicians were primarily physicians (7) with advanced practice clinicians (3) including physician assistants and nurse practitioners (Table 1).

**Table 1.** Clinician demographics by role.

	Gender male n	Age (median)	Visit count (median)
Clinician role (*n*)			
Physician (8)	6	41	385
Advanced practice (3)	1	44	206

### Qualitative results

Thematic results are described below and displayed with sample quotes (Table 2). The table with full length and additional quotes is available in the Supplementary material.

**Table 2.** Selected quotes by selected codes and theme.

Theme and code	Sample quote
Theme 1. Diagnosing pneumonia in the ED context is characterized by diagnostic uncertainty and may be a secondary priority relative to disposition and treatment	
1A. Diagnostic reasoning	*… I think for me, the big thing is really removing as much subjectivity out of the process as I can….* (Participant 1)
1B. Case diagnosis—diagnostic certainty—covering bases	*….I think oftentimes, we treat a lot of things empirically to cover in case it is pneumonia….* (Participant 7)
1C. Barriers to diagnostic accuracy	*She has like all the right risk factors for an aspiration pneumonia. I think the only thing I would maybe for future cases is, even if her X-ray was negative, looks good, I probably still would have acted as if this was such. I think a positive X-ray imaging helps support what you're advocating for the patient as a diagnosis, but a negative one doesn't necessarily rule out that there is no pneumonia. Yeah.* (Participant 4)
1D. Diagnostic ambiguity/uncertainty	*I think one that's interesting here is, … with some of these patients I would probably waffle a little bit and say, “No, this is my clinical impression,” ….*(Participant 7)
Theme 2. Existing diagnostic skill improvement processes are fragmented, inconsistent, and self-directed	
2A. Learning strategy—case review; feedback	*I followed his progress by looking up his chart…It's usually my own curiosity and chart stalking the patient…*(Participant 6)
2B. Learning strategy—experience	*Mistakes are unfortunate, but also, I think, one of the most potent teachers for a physician. You make a medical error, whether it's highly significant or not, that changes how you're going to approach things in the future.* (Participant 1)
2C. Learning strategy—case review	*Oftentimes, I'm completing notes. I'll be looking them up and following them, which I think is actually really, for me anyways, a great learning exercise.….* (Participant 5)
2D. Feedback	*A lot of times we do not, [get feedback] unless they come back as, what we call, a bounce back, or failure of treatment and coming back to be admitted to the hospital….*(Participant 4)
Theme 3. Clinicians liked the measure, feedback tool and features	
3A. User design reaction—reaction to normative data	*Doctors are….competitive and we like to know how we're doing compared to our colleagues because we want to see if we're doing okay….* (Participant 6)
3B. Tool evaluation—potential use	*You may make me more thoughtful about when I say about how I qualify things that we put into our diagnoses ….* (Participant 6)
3C. Tool evaluation—feedback	*reading through my note…you can tell that we weren't certain that she had a pneumonia, which is why I think we gave the diagnosis of possible pneumonia. In classic emergency medicine fashion, we kind of hedged….*(Participant 5)
Theme 4. Clinicians had strong reactions to feedback data across a spectrum from implicit trust in measure to extreme skepticism.	
4A. Interpretation—diagnostic discordance	*…. we like to think of pneumonia as a cut and dry diagnosis, or chest X-rays as black and white…. it's easier to call it pneumonia…* (Participant 3)
4B. Interpretation—data validity	*This would suggest I'm pretty [poor] at diagnosing pneumonia.* (Participant 2)
4C. User design reaction—measure	*[The comparison to my peers] is now just helping me understand, “Am I the worst person in the world? Do I need to find a totally different line of work or am I doing okay?”* (Participant 7)

#### THEME 1. Diagnosing pneumonia in the ED context is characterized by diagnostic uncertainty and may be a secondary priority relative to disposition and treatment

Clinicians described the diagnostic process for pneumonia in the ED as being fraught by uncertainty and ambiguity about the pneumonia diagnosis. Some clinicians described discomfort with diagnosis applied to ED settings, favoring the term diagnostic impressions given their uncertainty, “*I think one that's interesting here is, … with some of these patients I would probably waffle a little bit and say, ‘No, this is my clinical impression,’*” (Table 2, Quote 1D). Clinicians reported they valued diagnosis and constructed their workflow to improve accuracy (eg, to improve their information gathering or guard themselves against cognitive errors such as confirmation bias or diagnostic anchoring), “*I think for me, the big thing is really removing as much subjectivity out of the process as I can*.” (Table 2, Quote 1A). Many clinicians reported, however, that their primary goal in the ED was appropriate disposition and treatment of the patient and that the diagnosis was a vehicle to support treatment and admission to the hospital.

#### THEME 2. Existing diagnostic skill improvement processes are fragmented, inconsistent, and self-directed

Clinicians reported they valued and sought follow-up information on individual patients they were curious or worried about, “*I followed his progress by looking up his chart…It's usually my own curiosity and chart stalking the patient…*” (2A). Participants valued case review as a form of feedback, but they reported difficulty finding and tracking patients in their existing systems “*A lot of times we do not, [get feedback] unless they come back as, what we call, a bounce back, or failure of treatment and coming back to be admitted to the hospital….*” (2D).

#### THEME 3. Clinicians liked the measure, feedback tool, and features

Clinicians had a positive response to the performance measures (eg, positive predictive value), Venn Diagram, and information table of individual patient data. These data accurately reflected the clinicians’ patients and diagnoses, and the tool prompted memories of the specific patients. Clinicians appreciated task feedback (individual case accuracy and review), which sparked their interest in self-improvement “*You may make me more thoughtful about when I say about how I qualify things that we put into our diagnoses*” (3B). Normative feedback (comparison to peers) was consistent with clinicians’ self-reported values of excellence in performance and competition “*I think having a comparison with my colleagues is really beneficial because I kind of see where I am relative to them. I respect them*” (3D).

#### THEME 4. Clinicians had strong reactions to feedback data across a spectrum from implicit trust in measure to extreme skepticism

Implicit trust in the measure was reflected by concerns about participants’ own performance or suitability for their role “*[The comparison to my peers] is now just helping me understand, ‘Am I the worst person in the world? Do I need to find a totally different line of work or am I doing okay?’*” (4C). Skeptical reactions including direct challenges to the validity of concordance and/or the validity of the reference standards (chest imaging or discharge diagnosis), “*So if I diagnose someone with pneumonia, and I admit them to the hospital. And then, ultimately, on their discharge, they disagree, and they don't have a diagnosis of pneumonia, what further testing is gone in to reach that conclusion?*” (4D). Clinicians highlighted the nature of diagnosis as an evolving and ambiguous process, and a mismatch between this process and social pressures to assign patients to diagnostic labels and commit them to associated treatment pathways “*the discharge diagnosis looks strange to me…they said heart failure, and I'm not trying to pick apart another clinician's thing, but really what he had was acute hypoxemic respiratory failure*” (4E).

### Survey results

An overview of survey results is reported in Table 3. The median level of growth mindset in the sample was 4—representing a slight growth mindset (indicating a median of “somewhat disagree” with the statement that intelligence is stable). For failure attribution, the median value indicated a response for most failure-related questions between “believe none of the time” and “believe 25% of the time.” Comfort with feedback was between neutral and “somewhat comfortable.” The system usability scale score mean was 75, which is above the 68 considered “average” across systems.^61^

**Table 3.** Survey results.

Survey measure	Growth mindset	Failure attribution	Feedback comfort	System usability (SUS)
Median	4.33	0.8	3.33	75
Mean	4.44	0.93	3	71.67
Standard deviation	0.93	0.73	1.22	13.46
Scale range	2.33-5.33	0-10	2-5	60-92
*N*	9	9	9	9

**Figure 4.**
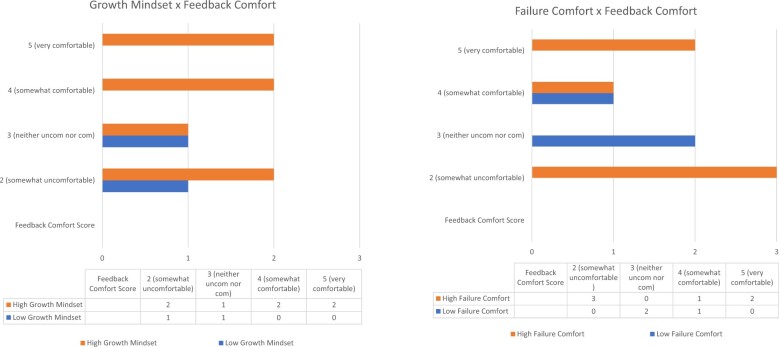
Individual beliefs and relationships to feedback comfort.

**Figure 5.**
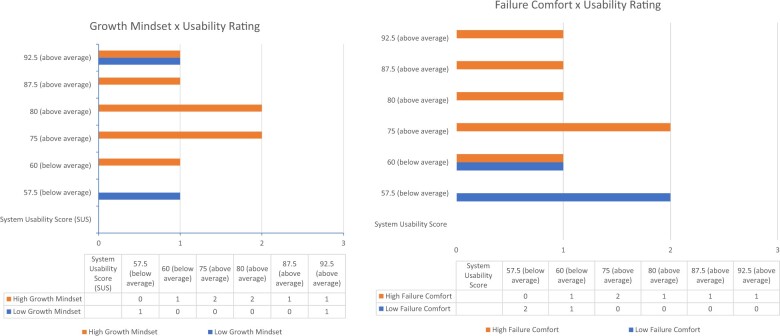
Individual beliefs and relationships to usability ratings.

The clinicians in our sample with beliefs consistent with a more fixed mindset (below-median ratings on the growth mindset scale) rated comfort with feedback lower than those with beliefs consistent with growth mindset (above median ratings) (Figure 4). Usability ratings were split: those with beliefs consistent with fixed mindset rated Dx-Connect far below or far above average on the SUS. Clinicians with low comfort with failure (*endorsing “do not believe” for most questions, below median ratings for our sample*) reported a restricted range of comfort receiving feedback *(neutral or somewhat comfortable)* whereas those with high comfort with failure responded across the range of the scale of feedback. Those with low comfort with failure rated the tool very low on usability (scores <61) whereas 5 of the 6 clinicians with higher comfort with failure rated the tool above average on usability (scores >70) (see Figure 5).

## Discussion

In an in-depth exploration of the process of diagnosis of pneumonia and clinician interaction with diagnostic performance feedback, ED clinicians reported valuing diagnostic feedback, enthusiasm for tools to provide feedback, and motivation to use feedback for practice improvement. Their characterization of diagnosis in the ED as a secondary priority to stabilization and treatment and strong responses to measures of discordances in diagnosis suggest complex relationships between measurement, feedback, and meaningful quality improvement in diagnosis for ED clinicians. This complexity requires a nuanced approach when developing paths to improvement. We found individual learning characteristics of mindset and failure attribution could influence clinicians’ reactions to feedback that warrants further study.

Our work has important implications for development and use of practice measures and feedback tools that target diagnostic accuracy. Diagnosis is an intuitive skill that should improve throughout a clinician’s experience. However, clinicians often plateau after training, because “for medical professionals to be able to keep improving their diagnostic performance during years of professional practice, they would need more feedback than the clinical environment naturally provides.”^62^ Clinicians in our study cited the importance of learning through experience but a lack of consistent, timely and accurate feedback that is required for improvement. Clinicians generally accept feedback when they believe it to be accurate.^63^ But clinicians in our study had mixed reactions to the feedback measures, ranging from acceptance to substantial disagreement, particularly when they were interpreted as evaluations of diagnostic performance or quality.

Measuring diagnostic accuracy is further challenged by the fact that the diagnosis of pneumonia lacks a gold standard and often carries substantial uncertainty, particularly in the ED, even though early and accurate diagnosis is recognized by clinicians as important. Diagnosis simultaneously represents both a clinician’s assessment of patient’s biophysical state and a tool for managing care processes to get a patient the treatment the clinician believes they need, even under uncertainty. In the ED, the assignment of a diagnosis is a method to communicate with other care teams and to justify appropriate treatment and evaluation while expeditiously moving the patient to the most appropriate site of care based on the clinicians’ overall clinical judgment, of patient status. Discordance between ED and chest imaging or discharge diagnoses could potentially represent cognitive errors, such as confirmation or anchoring bias, which can contribute to a diagnostic error.^64^^,^^65^

Our work suggests that for a pneumonia diagnosis with considerable uncertainty in the complex context of the ED, “diagnostic error” may be a mischaracterization of a measurement error. Our findings complement the clinician responses to a recent systematic review of diagnostic error in the ED conducted by the Agency for Health-Related Quality: a multiorganization response representing ED clinicians criticized the use of the term diagnostic error, emphasizing that “Emergency care is less about arriving at the final diagnosis, and more about real-time identification and treatment of life-threatening conditions.”^2^^,^^66^ This was similar to the impressions of the ED clinicians in our sample. In future work, it will be important to acknowledge uncertainty potentially by choosing labels for feedback that acknowledge this lack of gold standard rather than the “true positive” that we used in this display. Our study findings further highlight the challenge and importance of developing measures of diagnostic accuracy that adequately reflect the uncertainty of initial diagnoses and the importance of balancing accuracy with other aspects of high-quality emergency care.

Feedback has been highlighted as a missing link required to improve diagnostic performance, with new EHR capabilities providing new paths to improvement.^7^^,^^9^ Yet few tools exist in actual practice due to multiple sociotechnical barriers.^67^ Our study demonstrated the feasibility and usability of an EHR-based tool aimed at supporting diagnostic accuracy through feedback, and clinicians favorably assessed the tool. The intent of individual feedback in a culture of safety is to foster clinician curiosity, enhanced motivation to learn, and improvement in performance by using nonpunitive mechanisms.^7^ However, improvement through feedback is a complex and social process. Too great a focus on errors, comparison to peers, or provider incentives within a culture of blame historically endemic in medicine can result in biased feedback and interfere with the process of self-reflection and improvement.^68^ Previous performance measures in pneumonia have been criticized for lacking clear benefit to patients.^69^ As cultural and social processes in medicine evolve, understanding how provider and system experiences and attitudes toward feedback influence the use of measures are crucial to meaningful improvement in care.^70^^,^^71^

Our descriptive survey results suggested a potential relationship between learning mindset and individual responses to feedback and usability ratings, which has important implications for implementation and evaluation of feedback tools. Mindset theory and similar concepts have been proposed for use in medical education, clinical practice, quality improvement, and within models for data feedback systems.^18^^,^^19^^,^^24^^,^^72^ Part of the promise of this approach is that growth mindset can be influenced by interventions and could be harnessed to improve feedback experiences, promote useful and valuable feedback for clinicians, and shift paradigms of feedback toward a safety culture.^23^^,^^73^ Our survey results were limited to descriptive findings and will need confirmation in larger studies, but the theoretical basis for the work is an important strength.^74–76^

We recognize several limitations in our study. As a small study at a single institution, the results are not necessarily representative of clinician experiences at other ED environments. While our sample size is consistent with the classic Nielson usability sample rule and consistent with many other usability studies focused on clinicians,^60^ it is small. We were unable to systematically vary our study of reactions to specific feedback types (eg, normative vs specific task feedback) given our holistic approach to understanding reactions to Dx-Connect. Our preliminary study was not designed to investigate whether use of the tool improved diagnostic accuracy or skill in practice. We focused on clinician responses in qualitative interviews, so the influence of Dx-Connect feedback on practice and outcomes remains unknown and requires further study. Clinicians assessed the tool favorably but may have been influenced by being interviewed by members of the development team.^77^ Despite these limitations, our preliminary but intriguing findings suggest that performance feedback in diagnosis is feasible, usable, and perceived positively to clinicians as well as eliciting strong responses.

In the case of pneumonia where there is no gold standard for diagnostic accuracy, the impact of feedback about diagnostic performance is particularly complex and important to understand. When confronting clinicians with potentially imperfect measures and challenging feedback surrounding diagnoses, it is important to develop and use measures with humility, acknowledging uncertainty and the purpose of providing learning opportunities for professional growth.^78^ How should we incorporate the complexities of the diagnostic context within feedback processes? How can we ensure feedback is provided in a supportive way to maximize the likelihood of a constructive process of sensemaking and improvement? These are critical questions pointing to future directions for promoting diagnostic excellence for complex diseases.

## Supplementary Material

ocae112_Supplementary_Data

## Data Availability

Survey data are incorporated into the article. Electronic Health Record Data cannot be released for ethical and privacy reasons.

## References

[1] Leape LL , Brennan TA, Laird N, et al The nature of adverse events in hospitalized patients. Results of the Harvard Medical Practice Study II. N Engl J Med. 1991;324(6):377-384. 10.1056/NEJM199102073240605. 1824793

[2] Newman-Toker DE , Peterson SM, Badihian S, et al AHRQ Comparative Effectiveness Reviews. Diagnostic Errors in the Emergency Department: A Systematic Review. Agency for Healthcare Research and Quality (US); 2022. 36574484

[3] Meyer AND , Upadhyay DK, Collins CA, et al A program to provide clinicians with feedback on their diagnostic performance in a learning health system. Jt Comm J Qual Patient Saf. 2021;47(2):120-126. 10.1016/j.jcjq.2020.08.014. 32980255

[4] McGlynn EA , McDonald KM, Cassel CK. Measurement is essential for improving diagnosis and reducing diagnostic error: a report from the Institute of Medicine. JAMA. 2015;314(23):2501-2502. 10.1001/jama.2015.13453. 26571126

[5] AHRQ. Diagnostic safety and quality. 2022. Accessed May 15, 2024. https://www.ahrq.gov/topics/diagnostic-safety-and-quality.html

[6] Fernandez Branson C , Williams M, Chan TM, et al Improving diagnostic performance through feedback: the Diagnosis Learning Cycle. BMJ Qual Saf. 2021;30(12):1002-1009. 10.1136/bmjqs-2020-012456. PMC8606468 34417335

[7] Larson DB , Donnelly LF, Podberesky DJ, Merrow AC, Sharpe RE Jr, Kruskal JB. Peer feedback, learning, and improvement: answering the call of the institute of medicine report on diagnostic error. Radiology. 2017;283(1):231-241. 10.1148/radiol.2016161254. 27673509

[8] Khumrin P , Ryan A, Juddy T, Verspoor K. DrKnow: a diagnostic learning tool with feedback from automated clinical decision support. AMIA Annu Symp Proc. 2018 2018:1348-1357. 30815179 PMC6371235

[9] Vasey B , Ursprung S, Beddoe B, et al Association of clinician diagnostic performance with machine learning-based decision support systems: a systematic review. JAMA Netw Open. 2021;4(3):e211276. 10.1001/jamanetworkopen.2021.1276. 33704476 PMC7953308

[10] Croskerry P. Advances in patient safety diagnostic failure: a cognitive and affective approach In: Henriksen K, Battles JB, Marks ES, Lewin DI, eds. Advances in Patient Safety: From Research to Implementation (Volume 2: Concepts and Methodology). Agency for Healthcare Research and Quality (US); 2005. 21249825

[11] DiCuccio MH. The relationship between patient safety culture and patient outcomes: a systematic review. J Patient Saf. 2015;11(3):135-142. 10.1097/pts.0000000000000058. 24583952

[12] Cochrane BS , Hagins M, Picciano G, et al High reliability in healthcare: creating the culture and mindset for patient safety. Healthc Manage Forum. 2017;30(2):61-68. 10.1177/0840470416689314. 28929881

[13] Croskerry P. Diagnostic failure: a cognitive and affective approach. In: Henriksen K, Battles JB, Marks ES, Lewin DI, eds. Advances in Patient Safety: From Research to Implementation (Volume 2: Concepts and Methodology). Agency for Healthcare Research and Quality (US); 2005. 21249825

[14] Croskerry P. The feedback sanction. Acad Emerg Med. 2000;7(11):1232-1238. 10.1111/j.1553-2712.2000.tb00468.x. 11073471

[15] Institute of Medicine (U.S.). New Frontiers in Patient Safety. National Academies Press; 2011.

[16] Institute of Medicine Committee on Quality of Health Care in America. To Err is Human: Building a Safer Health System (Kohn LT, Corrigan JM, Donaldson MS, eds.). National Academies Press (US); 2000. 25077248

[17] Patel S , Pierce L, Jones M, et al Using participatory design to engage physicians in the development of a provider-level performance dashboard and feedback system. Jt Comm J Qual Patient Saf. 2022;48(3):165-172. 10.1016/j.jcjq.2021.10.003. 35058160 PMC8885889

[18] Grimes C. The BMJ Opinion 2020. [cited 2022]. Accessed October 20, 2023. https://blogs.bmj.com/bmj/2020/08/15/caris-grimes-clinicians-need-to-learn-how-to-manage-failure/

[19] Klein J , Delany C, Fischer MD, Smallwood D, Trumble S. A growth mindset approach to preparing trainees for medical error. BMJ Qual Saf. 2017;26(9):771-774. 10.1136/bmjqs-2016-006416. 28400404

[20] Whitelock-Wainwright E , Koh JW, Whitelock-Wainwright A, Talic S, Rankin D, Gašević D. An exploration into physician and surgeon data sensemaking: a qualitative systematic review using thematic synthesis. BMC Med Inform Decis Mak. 2022;22(1):256. 10.1186/s12911-022-01997-1. 36171583 PMC9520820

[21] Weick KE. Sensemaking in Organizations. Sage Publications; 1995.

[22] Rattan A , Savani K, Naidu NVR, Dweck CS. Can everyone become highly intelligent? Cultural differences in and societal consequences of beliefs about the universal potential for intelligence. J Pers Soc Psychol. 2012;103(5):787-803. 10.1037/a0029263. 22800285

[23] Dweck CS , Yeager DS. A growth mindset about intelligence In: Walton GM, Crum AJ, eds. Handbook of Wise Interventions: How Social Psychology Can Help People Change. The Guilford Press; 2021:9-35.

[24] Richardson D , Kinnear B, Hauer KE, ICBME Collaborators, et al Growth mindset in competency-based medical education. Med Teach. 2021;43(7):751-757. 10.1080/0142159x.2021.1928036. 34410891

[25] Nussbaum AD , Dweck CS. Defensiveness versus remediation: self-theories and modes of self-esteem maintenance. Pers Soc Psychol Bull. 2008;34(5):599-612. 10.1177/0146167207312960. 18276895

[26] Yeager DS , Dweck CS. What can be learned from growth mindset controversies? Am Psychol. 2020;75(9):1269-1284. 10.1037/amp0000794. 33382294 PMC8299535

[27] Haimovitz K , Dweck CS. What predicts children’s fixed and growth intelligence mind-sets? Not their parents’ views of intelligence but their parents’ views of failure. Psychol Sci. 2016;27(6):859-869. 10.1177/0956797616639727. 27113733

[28] Conroy DE , Pincus AL. Interpersonal impact messages associated with different forms of achievement motivation. J Pers. 2011;79(4):675-706. 10.1111/j.1467-6494.2011.00693.x. 21682724

[29] Conroy DE , Willow JP, Metzler JN. Multidimensional fear of failure measurement: the Performance Failure Appraisal Inventory. J Appl Sport Psychol. 2002;14(2):76-90. 10.1080/10413200252907752.

[30] Wright AGC , Pincus AL, Conroy DE, Elliot AJ. The pathoplastic relationship between interpersonal problems and fear of failure. J Pers. 2009;77(4):997-1024. 10.1111/j.1467-6494.2009.00572.x. 19558445

[31] Knowles MS , Holton EF III, Swanson RA. The Adult learner: The Definitive Classic in Adult Education and Human Resource Development. Routledge; 2014.

[32] Cavallazzi R , Furmanek S, Arnold FW, et al The burden of community-acquired pneumonia requiring admission to ICU in the United States. Chest. 2020;158(3):1008-1016. 10.1016/j.chest.2020.03.051. 32298730 PMC9458541

[33] Mortensen EM , Coley CM, Singer DE, et al Causes of death for patients with community-acquired pneumonia: results from the pneumonia patient outcomes research team cohort study. Arch Intern Med. 2002;162(9):1059-1064. 10.1001/archinte.162.9.1059. 11996618

[34] Sarode VR , Datta BN, Banerjee AK, et al Autopsy findings and clinical diagnoses: a review of 1,000 cases. Hum Pathol. 1993;24(2):194-198. 10.1016/0046-8177(93)90300-6. 8432514

[35] Yayci N , Pakis I, Karapirli M, Celik S, Uysal C, Polat O. The review of autopsy cases of accidental childhood deaths in Istanbul. J Forensic Leg Med. 2011;18(6):253-256. 10.1016/j.jflm.2011.04.009. 21771555

[36] Basi SK , Marrie TJ, Huang JQ, Majumdar SR. Patients admitted to hospital with suspected pneumonia and normal chest radiographs: epidemiology, microbiology, and outcomes. Am J Med. 2004;117(5):305-311. 10.1016/j.amjmed.2004.03.029. 15336579

[37] Jones BE , Jones J, Bewick T, et al CURB-65 pneumonia severity assessment adapted for electronic decision support. Chest. 2011;140(1):156-163. 10.1378/chest.10-1296. 21163875 PMC3198487

[38] Daniel P , Bewick T, Welham S, McKeever TM, Lim WS, for the British Thoracic Society. Adults miscoded and misdiagnosed as having pneumonia: results from the British Thoracic Society pneumonia audit. Thorax. 2017;72(4):376-379. 10.1136/thoraxjnl-2016-209405. 28108620

[39] Mate K , Fulmer T, Pelton L, et al Evidence for the 4Ms: interactions and outcomes across the care continuum. J Aging Health. 2021;33(7-8):469-481. 10.1177/0898264321991658. 33555233 PMC8236661

[40] Wang AH , Newman K, Martin LS, Lapum J. Beyond instrumental support: mobile application use by family caregivers of persons living with dementia. Dementia (London). 2022;21(5):1488-1510. 10.1177/14713012211073440. 35414298 PMC9237854

[41] Joint Commission Resources Inc. Risk Assessment for Infection Prevention and Control. Joint Commission Resources; 2010.

[42] Frisbee J , Heidel RE, Rasnake MS. Adverse outcomes associated with potentially inappropriate antibiotic use in heart failure admissions. Open Forum Infect Dis. 2019;6(6):ofz220. 10.1093/ofid/ofz220. 31211161 PMC6559271

[43] Gergenti L , Olympia RP. Etiology and disposition associated with radiology discrepancies on emergency department patients. Am J Emerg Med. 2019;37(11):2015-2019. 10.1016/j.ajem.2019.02.027. 30799026

[44] Long B , Long D, Koyfman A. Emergency medicine evaluation of community-acquired pneumonia: history, examination, imaging and laboratory assessment, and risk scores. J Emerg Med. 2017;53(5):642-652. 10.1016/j.jemermed.2017.05.035. 28941558

[45] Kuhn J , van den Berg P, Mamede S, Zwaan L, Bindels P, van Gog T. Improving medical residents' self-assessment of their diagnostic accuracy: does feedback help? Adv Health Sci Educ Theory Pract. 2022;27(1):189-200. 10.1007/s10459-021-10080-9. 34739632 PMC8938348

[46] Jones BE , South BR, Shao Y, et al Development and validation of a natural language processing tool to identify patients treated for pneumonia across VA emergency departments. Appl Clin Inform. 2018;9(1):122-128. 10.1055/s-0038-1626725. 29466818 PMC5821510

[47] Chapman AB , Peterson KS, Rutter E, et al Development and evaluation of an interoperable natural language processing system for identifying pneumonia across clinical settings of care and institutions. JAMIA Open. 2022;5(4):ooac114. 10.1093/jamiaopen/ooac114. 36601365 PMC9801965

[48] Hoffman RR , Militello LG. Perspectives on Cognitive Task Analysis: Historical Origins and Modern Communities of Practice. Psychology Press; 2008.

[49] Crandall B , Klein GA, Hoffman RR. Working Minds: A Practitioner's Guide to Cognitive Task Analysis. MIT Press; 2006.

[50] Dweck CS. Mindset: The New Psychology of Success. New York, NY: Random House; 2006.

[51] Dweck CS. Self-Theories: Their Role in Motivation, Personality, and Development. New York, NY: Psychology Press; 1999.

[52] Conroy DE , Metzler JN. Temporal stability of performance failure appraisal inventory items. Measur Phys Educ Exercise Sci. 2003;7(4):243-261. 10.1207/S15327841MPEE0704_3.

[53] Brooke J. SUS: A quick and dirty usability scale. Usability Eval Ind. 1995;189-194.

[54] Lewis JR. The System Usability Scale: past, present, and future. Int J Human-Comput Interact. 2018;34(7):577-590. 10.1080/10447318.2018.1455307.

[55] Patton MQ. Qualitative Research & Evaluation Methods. 3rd ed. Sage Publications, Inc; 2002.

[56] Braun V , Clarke V, Hayfield N, Terry G. Thematic analysis. In: Liamputtong P, ed. Handbook of Research Methods in Health Social Sciences. Springer; 2019:843-860.

[57] QIP Ltd. NVivo. 2020. Accessed June 1, 2023.

[58] Morse JM. Critical analysis of strategies for determining rigor in qualitative inquiry. Qual Health Res. 2015;25(9):1212-1222. 10.1177/1049732315588501. 26184336

[59] Dedoose. Web Application for Managing, Analyzing, and Presenting Qualitative and Mixed Method Research Data, Version 9.0.17. SocioCultural Research Consultants, LLC; 2021.

[60] Nielsen J. Usability Engineering. Academic Press; 1993.

[61] Lewis J , Sauro J. Item benchmarks for the system usability scale. *Journal of User Experience*. 2018;13:158-167.

[62] Ericsson KA. Deliberate practice and the acquisition and maintenance of expert performance in medicine and related domains. Acad Med. 2004;79(10 Suppl):S70-81. 10.1097/00001888-200410001-00022. 15383395

[63] Eden AR , Hansen E, Hagen MD, Peterson LE. Physician perceptions of performance feedback in a quality improvement activity. Am J Med Qual. 2018;33(3):283-290. 10.1177/1062860617738327. 29088919

[64] Pines JM. Profiles in patient safety: confirmation bias in emergency medicine. Acad Emerg Med. 2006;13(1):90-94. 10.1197/j.aem.2005.07.028. 16365325

[65] Blumenthal-Barby JS , Krieger H. Cognitive biases and heuristics in medical decision making: a critical review using a systematic search strategy. Med Decis Making. 2015;35(4):539-557. 10.1177/0272989x14547740. 25145577

[66] ACEP. EM organizations issue letter regarding AHRQ Report on diagnostic errors in the ED. 2022. Accessed May 16, 2024. https://www.acep.org/news/acep-newsroom-articles/acep-em-organizations-issue-letter-regarding-ahrq-report-on-diagnostic-errors-in-the-ed/

[67] Rosner BI , Zwaan L, Olson APJ. Imagining the future of diagnostic performance feedback. Diagnosis (Berl). 2022;10(1):31-37. 10.1515/dx-2022-0055. 36378520

[68] LaDonna KA , Ginsburg S, Watling C. Shifting and sharing: academic physicians' strategies for navigating underperformance and failure. Acad Med. 2018;93(11):1713-1718. 10.1097/acm.0000000000002292. 29794519

[69] Mitchell I , Schuster A, Smith K, Pronovost P, Wu A. Patient safety incident reporting: a qualitative study of thoughts and perceptions of experts 15 years after 'To Err is Human’. BMJ Qual Saf. 2016;25(2):92-99. 10.1136/bmjqs-2015-004405. 26217037

[70] Scott P , Dunscombe R, Evans D, Mukherjee M, Wyatt J. Learning health systems need to bridge the ‘two cultures’ of clinical informatics and data science. J Innov Health Inform. 2018;25(2):126-131. 10.14236/jhi.v25i2.1062. 30398454

[71] Manojlovich M , Hofer TP, Krein SL. Advancing patient safety through the clinical application of a framework focused on communication. J Patient Saf. 2018;17(8):e732-e737. 10.1097/pts.0000000000000547. 30383622

[72] Klein J , McColl G. Cognitive dissonance: how self-protective distortions can undermine clinical judgement. Med Educ. 2019;53(12):1178-1186. 10.1111/medu.13938. 31397007

[73] DeBacker TK , Heddy BC, Kershen JL, Crowson HM, Looney K, Goldman JA. Effects of a one-shot growth mindset intervention on beliefs about intelligence and achievement goals. Educational Psychology. 2018;38(6):711-733. 10.1080/01443410.2018.1426833

[74] Coiera E , Ammenwerth E, Georgiou A, Magrabi F. Does health informatics have a replication crisis? J Am Med Inform Assoc. 2018;25(8):963-968. 10.1093/jamia/ocy028. 29669066 PMC6077781

[75] Proulx T , Morey RD. Beyond statistical ritual: theory in psychological science. Perspect Psychol Sci. 2021;16(4):671-681. 10.1177/17456916211017098. 34240651

[76] Ancker JS , Benda NC, Reddy M, Unertl KM, Veinot T. Guidance for publishing qualitative research in informatics. J Am Med Inform Assoc. 2021;28(12):2743-2748. 10.1093/jamia/ocab195. 34537840 PMC8633663

[77] Dell N , Vaidyanathan V, Medhi I, Cutrell E, Thies W. "Yours is better!": participant response bias in HCI. In: Proceedings of the SIGCHI Conference on Human Factors in Computing Systems. Association for Computing Machinery; 2012: 1321-1330.

[78] Harrison J , Klein J. ‘Remember that patient you saw…': advice for trainees on coping after making an error. Emerg Med Australas. 2019;31(4):667-668. 10.1111/1742-6723.13347. 31287211

