# Supplementary material for: Pneumonia diagnosis performance in the emergency department: a mixed-methods study about clinicians’ experiences and exploration of individual differences and response to diagnostic performance feedback
Source: J Am Med Inform Assoc. 2024 May 25;31(7):1503–13. doi: 10.1093/jamia/ocae112 (PMC11187426; doi:10.1093/jamia/ocae112)
Supplement: ocae112_Supplementary_Data [file ocae112_supplementary_data.zip › ocae112_Supplementary_Data/Supplementary File_InterviewScript.docx]

**Semi-Structured Interview Guide**

**INTRODUCTION: (+0 min)**

We are researchers working at the University of Utah to develop a feedback report. The report is intended to support physician development of diagnostic skills. For our use case, we are looking at pneumonia because it is a common clinical problem for front-line physicians but lacks a diagnostic gold standard and the diagnosis of pneumonia often changes across a patient’s hospitalization-- about 50 percent of the time.

We want to know if this report helps physicians review their patient’s diagnostic story and supports learning. We are looking for your feedback as an expert clinician.

Have you read the consent letter that was emailed to you? Do you have questions?

We are interested in your personal experiences and there are no right or wrong responses to our questions. We are not assessing your skill or knowledge in any way. We want to understand what it is like in the real world.

We will record this interview which has several parts. The first part is a summary of a patient care episode and discussion of the diagnostic process. In the second we will ask for your feedback on the design of a diagnostic report, and afterward there is a questionnaire exploring its validity and usefulness. We estimate that the interview will take about 50 minutes. Your participation is voluntary. You can stop at any time or decline to answer any question.

Do you agree to participate? Thank you! I will start the recording now.

**Part 1a: PROCESS OF DIAGNOSIS (Cognitive task analysis) (+7 min)**

1. “In the past quarter, about how many patients do you think you diagnosed with possible pneumonia?
2. Can you take a moment to think of one particular patient?
3. When you are ready, please give a **1-minute** overview of the patient as though you were describing the case to a colleague.

**Part 1b: ELICIT DETAIL (+12 min)**

**The next questions will help us to understand the process of diagnosis in the ED.**

1. When you first saw the patient, what was your main concern?
2. Did the patient remind you of any previous cases?
   1. How did that affect your tests, dx, treatment?
3. Did you communicate the diagnosis to other providers/staff?
   1. If yes - Was there dialogue or disagreement? - How did you explain your reasoning?
4. *What about the diagnosis made it particularly difficult or memorable?*
5. *Did anything happen with the patient that surprised you?*
6. *What did you notice that led you to first suspect pneumonia?*
7. *How certain were you about the diagnosis? Why?*

**Part 1c: PROCESS OF SEEKING OR RECEIVING FEEDBACK (+17 min)**

1. Did you ever find out what happened to the patient?
2. If yes, **why** did you get that information?
3. In general, how do you get feedback about your diagnostic accuracy?
4. What causes you to remember patients?

**Part 1d: STEPS TO IMPROVE SKILLS (+20 min)**

**The next questions will help us to understand how ED providers may seek to improve their diagnostic accuracy.**

1. If you wanted to increase your skills in diagnosis, what would you do?
   1. What strategies have you tried that have worked/not worked?
2. Do you review your patient’s records to improve your diagnostic skills?
3. How do you feel that you learn best about disease presentation and diagnosis?
4. How do you think that most physicians learn?

**Part 2a. FEEDBACK DISPLAY (+23 min)**

Next, you will see a report of pneumonia patients who you saw and admitted at University of Utah Hospital. To help us assess the usability of the design, as you look at the report can you please describe what you see?

<Prompt if needed to elicit feedback for major sections. If questions are asked rather than provide an answer say, “what does it appear to be” or “Would you except it to be?” or “Is that what you expect it to be?”>

1. What do you see? What does it mean?

1) Venn Diagram

2) measures

3) Department Results

4) Patient list

1. What could you do with this information?

**PART 2b: DIAGNOSTIC DISCORDANCE (Allow user to view notes) (+35 min)**

**Now we would like to understand if the information in the patient list is meaningful.**

1. Can you remember seeing any of the patients on this list?
   1. If yes, how many?
      1. What elements helped you remember the case?
   2. If no, what additional information would you need to remember the patient?
2. Please select one of the patients with a diagnostic discordance.
   1. Are the discharge and chest imaging diagnoses what you might have expected?
   2. Can you explain the source of the discordance? (attribution)
   3. How might this information effect your diagnosis in the future? (self-determination, motivation)
3. Select a patient with consistent agreement across the diagnosis.
4. Are the discharge and chest imaging diagnoses what you might have expected?
5. How might this information effect your diagnosis in the future?
6. Can you explain the reason for agreement?

**PART 2c: VALUE RATINGS** **(+50 min)**

**We would like your opinion of the value of this report.**

1. Overall, how useful is this report from 1-Very useful to 5-Not very useful?
   1. Why?
2. Please give me a rating of usefulness of each section from 1- Very useful to 5- not very useful for each of the sections:
   1. Venn Diagram
   2. Performance Measures
   3. Patient List Specifics
   4. Access to the provider notes
   5. Department measures page
3. What information could be removed?
4. What is missing?
5. Do you have any other comments or suggestions?

**Part 3: SURVEY (+55 min)**

We have placed a link in the chat. Please copy the link to take a short survey.

- <https://utah.sjc1.qualtrics.com/jfe/form/SV_5AywFKsCBJmd2Xs>

**Conclusion**

Thank you for participating. Your feedback is very helpful.
