# Supplementary material for: Pneumonia diagnosis performance in the emergency department: a mixed-methods study about clinicians’ experiences and exploration of individual differences and response to diagnostic performance feedback
Source: J Am Med Inform Assoc. 2024 May 25;31(7):1503–13. doi: 10.1093/jamia/ocae112 (PMC11187426; doi:10.1093/jamia/ocae112)
Supplement: ocae112_Supplementary_Data [file ocae112_supplementary_data.zip › ocae112_Supplementary_Data/Table3_21_2024_cleanLONG.docx]

**Table 2.** Selected Quotes by Selected Codes and Theme

| **Theme and Code** | **Sample Quote** |
| --- | --- |
| **Theme 1. Diagnosing Pneumonia in the ED Context is characterized by diagnostic uncertainty and may be a secondary priority relative to disposition and treatment.** | |
| 1A. Diagnostic Reasoning | *…* *I think for me, the big thing is really removing as much subjectivity out of the process as I can….* (Participant 1) |
| 1B. Case Diagnosis – Diagnostic Certainty – Covering Bases | *….I think oftentimes, we treat a lot of things empirically to cover in case it is pneumonia….*(Participant 7) |
| 1C. Barriers to Diagnostic Accuracy | *She has like all the right risk factors for an aspiration pneumonia. I think the only thing I would maybe for future cases is, even if her X-ray was negative, looks good, I probably still would have acted as if this was such. I think a positive X-ray imaging helps support what you're advocating for the patient as a diagnosis, but a negative one doesn't necessarily rule out that there is no pneumonia. Yeah. .*(Participant 4) |
| 1D. Barriers to Diagnostic Accuracy | *The patient still remained pretty ambiguous, never really showed any obvious sepsis physiology…..*(Participant 7) |
| 1E. Barriers to Diagnostic Accuracy | *…. there's really no gold standard for this. And I feel like a lot of us in medicine are strongly desire to put a label on something for a patient. And I think oftentimes, that burden falls more on these inpatient teams who are kind of required to make a discharge diagnosis, even when there may not be a clear one. And so I'm, I'm always kind of interested in how easily we slap a label on a patient just to do that, because the patient wants it or because we have to, to put it on a discharge diagnosis. And so I still wonder if there were a gold standard for diagnosis, what the gold standard are, you know, what the numbers would look like for those discharge diagnoses?* (Participant 4) |
| 1F. Diagnostic ambiguity/uncertainty | *I think one that's interesting here is, … with some of these patients I would probably waffle a little bit and say, "No, this is my clinical impression," ….*(Participant 7) |
| **Theme 2. Existing diagnostic skill improvement processes are fragmented, inconsistent, and self-directed** | |
| 2A. Learning strategy – case review; feedback | *I followed his progress by looking up his chart…It's usually my own curiosity and chart stalking the patient…*(Participant 6) |
| 2B. Learning strategy – experience | *Mistakes are unfortunate, but also, I think, one of the most potent teachers for a physician. You make a medical error, whether it's highly significant or not, that changes how you're going to approach things in the future.* (Participant 1) |
| 2C. Learning strategy – case review | *Oftentimes, I'm completing notes. I'll be looking them up and following them, which I think is actually really, for me anyways, a great learning exercise.….* (Participant 5) |
| 2D. Feedback – | *A lot of times we do not, [get feedback] unless they come back as, what we call, a bounce back, or failure of treatment and coming back to be admitted to the hospital….*(Participant 4) |
| **Theme 3. Clinicians liked the measure, feedback tool and features** | |
| 3A. User Design Reaction – Reaction to Normative Data | *Doctors are….competitive and we like to know how we're doing compared to our colleagues because we want to see if we're doing okay….* (Participant 6) |
| 3B. Tool evaluation – potential use | *You may make me more thoughtful about when I say about how I qualify things that we put into our diagnoses ….* (Participant 6) |
| 3C. Tool evaluation – feedback | *reading through my note…you can tell that we weren't certain that she had a pneumonia, which is why I think we gave the diagnosis of possible pneumonia. In classic emergency medicine fashion, we kind of hedged….*(Participant 5) |
| 3D. User design reactions – measure | *I think having a comparison with my colleagues is really beneficial because I kind of see where I am relative to them. I respect them….* (Participant 1) |
| 3E. User design reactions – measure | *That would be the-- to get better would be the goal here. But I personally think this kind of information is always helpful….*(Participant 6) |
| **Theme 4. Clinicians had strong reactions to feedback data across a spectrum from implicit trust in measure to extreme skepticism.** | |
| 4A. Interpretation – Diagnostic Discordance | *…. we like to think of pneumonia as a cut and dry diagnosis, or chest X-rays as black and white…. it's easier to call it pneumonia…* (Participant 3) |
| 4B. Interpretation – data validity | *This would suggest I'm pretty [poor] at diagnosing pneumonia.*  (Participant 2) |
| 4C. User design reaction – measure | *[The comparison to my peers] is now just helping me understand, "Am I the worst person in the world? Do I need to find a totally different line of work or am I doing okay?"* (Participant 7) |
| 4D. Interpretation – data validity | *“So if I diagnose someone with pneumonia, and I admit them to the hospital. And then, ultimately, on their discharge, they disagree, and they don't have a diagnosis of pneumonia, what further testing is gone in to reach that conclusion? Because the testing that we're capable of doing and the testing that happens while someone is inpatient is not the same.”* (Participant 1) |
| 4E. Data Reaction – Validity | *…the discharge diagnosis looks strange to me…they said heart failure, and I'm not trying to pick apart another clinician's thing, but really what he had was acute hypoxemic respiratory failure…*  (Participant 3) |
| 4F. Interpretation – data | *I'll just say many, many such an excellent learning tool has been turned into outcomes evaluation, and skills evaluation and [performance] scores…….* (Participant 2) |
